# A classroom-based multisensory ELT method for observational documentation of LSRW skills

**DOI:** 10.1016/j.mex.2026.104097

**Published:** 2026-08-15

**Authors:** Srini Sandhya M, B.R. Aravind

**Affiliations:** a Research Scholar, Department of English, Kalasalingam Academy of Research and Education, Tamil Nadu, India; b Head & Assistant Professor, Department of English, Kalasalingam Academy of Research and Education, Tamil Nadu, India

**Keywords:** Multisensory instruction, English language teaching, Classroom-based assessment, Observational rubric, Early primary learners, LSRW skills

## Abstract

This paper presents the Multisensory LSRW Classroom Observation Method (M-LCOM) for documenting listening, speaking, reading, and writing (LSRW) behaviours in early primary learners during routine classroom instruction. The method addresses practical limitations of assessment in school-based contexts, where standardised testing is often infeasible due to time constraints, classroom disruption, and developmental considerations. The approach integrates routine instructional practice with real-time classroom observation through a phase-based 40-hour programme organised across four LSRW domains. Learner behaviours are documented using predefined behavioural indicators and a three-level developmental rubric (Emerging, Developing, Secure), enabling consistent observation without interrupting instructional flow. The method conceptualises learning as task-related behaviour demonstrated during instructional engagement and provides procedural guidelines for instructional sequencing, observation boundaries, scoring procedures, and spreadsheet-based data recording. Designed for feasibility, ecological validity, and replicability, the method offers a practical framework for documenting language learning behaviours in low-resource ELT contexts.

Method details:

• Enables real-time, classroom-based documentation of LSRW skills using predefined behavioural indicators and a three-level developmental rubric
• Integrates routine multisensory instructional activities with classroom observation, eliminating the need for separate assessment sessions
• Provides a replicable and low-resource methodological framework using standard classroom materials and spreadsheet-based data recording.

## Graphical abstract

**Figure:**
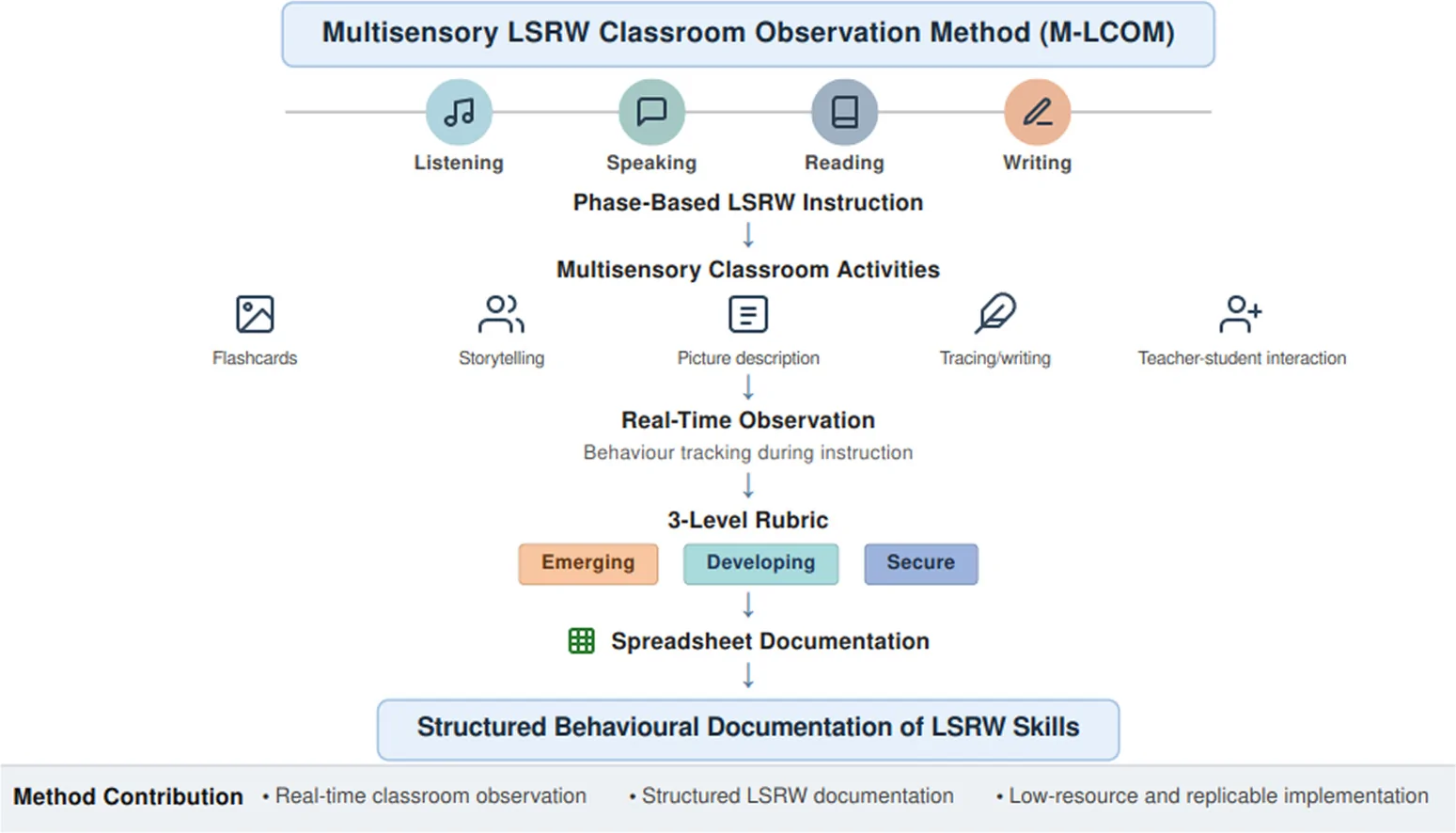

**Specifications table**

**Table:** 

Subject area	Psychology
**More specific subject area**	English language teaching (ELT)
**Name of your method**	Multisensory LSRW Classroom Observation Method
**Name and reference of original method**	Not applicable (custom classroom-based method)
**Resource availability**	Standard classroom materials (flashcards, worksheets, visual aids); spreadsheet-based data recording (e.g., Microsoft Excel); no specialised software required.

**Resource Availability**

The method requires only standard classroom materials, including flashcards, worksheets, and visual aids, which are commonly available in early primary ELT settings. Observational data are recorded using spreadsheet software (e.g., Microsoft Excel), with predefined columns for learner identifiers, LSRW domains, and observation time points.

The observation framework is based on predefined behavioural indicators and a three-level developmental rubric (Emerging, Developing, Secure). All components of the method—including instructional sequencing, observation procedures, scoring criteria, and data recording formats—are described in sufficient detail within the manuscript to enable replication in similar classroom contexts.

## Background

In early primary English Language Teaching (ELT) contexts, assessment is often constrained by practical limitations, including time [1], classroom disruption, and the developmental appropriateness of formal testing procedures [2,3]. As a result, classroom-based evaluation frequently relies on informal teacher observations or loosely structured documentation practices, which, although widely used, lack consistency, transparency, and methodological rigor [3]. This creates a gap between everyday instructional practices and consistent documentation of learner behaviours.

Multisensory and multimodal approaches are widely embedded in early ELT classrooms, where learning is mediated through visual, auditory, and embodied interaction [4,5]. From a multimodal perspective, meaning-making is distributed across multiple semiotic modes rather than confined to linguistic input alone, allowing young learners to engage with language through diverse sensory channels [4,6]. Empirical research has further demonstrated that such multimodal and multisensory instructional practices support engagement and early language development, particularly in young learners [5,7]. However, while these approaches are pedagogically well established, their role in supporting classroom observation and documentation of learner behaviours remains underexplored.

From a sociocultural standpoint, learning is conceptualised as a socially mediated and interaction-driven process, where observable behaviours during task engagement reflect underlying cognitive development [29]. Classroom interaction, participation, and responsiveness are therefore not merely instructional outcomes but also meaningful indicators of learning processes [8]. This perspective aligns closely with formative assessment frameworks, which emphasise continuous, embedded evaluation within instructional contexts rather than discrete testing events [1,9,31].

Despite the theoretical alignment between multisensory instruction, sociocultural learning, and formative assessment, existing assessment practices in early ELT remain predominantly outcome-oriented or test-based [10]. Even classroom-based assessment approaches often lack structured observational frameworks that can be consistently applied across instructional contexts [10,11]. Recent research has highlighted the increasing role of digital technologies in supporting classroom-based documentation and instructional consistency; however, these tools are typically positioned as instructional aids rather than methodological supports for classroom observation [12,13].

Furthermore, while structured classroom observation protocols have been developed in broader educational research contexts, demonstrating the feasibility of real-time behavioural coding and systematic data collection [14,15,26], such approaches have not been sufficiently adapted to early ELT contexts, particularly in low-resource classroom environments [16]. This limits teachers’ and researchers’ ability to generate replicable, ecologically valid documentation of language learner behaviours in authentic settings.

In response to these gaps, the present study proposes a classroom-based multisensory ELT method that integrates instructional practice with structured, rubric-based observation of listening, speaking, reading, and writing (LSRW) behaviours. Rather than serving as an intervention or a standardised assessment tool, the method provides a structured framework for documenting learner behaviours during routine classroom instruction. By embedding classroom observation into instructional processes, the approach aims to enhance transparency, consistency, and replicability in early ELT classroom assessment, particularly in low-resource educational contexts [9].

## Method details

### Overview of the method

This paper presents the Multisensory LSRW Classroom Observation Method (M-LCOM) designed to enable documentation of classroom learning behaviours in listening, speaking, reading, and writing (LSRW) among early primary learners. The method integrates routine instructional activities with real-time observational scoring, thereby eliminating the need for separate assessment sessions or standardised testing conditions. This design aligns with classroom-based formative assessment principles, where evaluation is embedded within instructional processes [9].

Unlike outcome-oriented assessment models, the present method conceptualises learning as task-related behaviour demonstrated during instructional activity. This perspective is consistent with sociocultural approaches to learning, which emphasise interaction, participation, and responsiveness as indicators of developmental processes [29]. The method, therefore, prioritises ecological validity by capturing learning behaviours within authentic classroom contexts.

### Instructional structure

The instructional component consists of a structured 40-hour programme implemented over eight weeks. Teaching is organised into four sequential phases corresponding to the core LSRW domains: Listening, Speaking, Reading, and Writing.

Each phase incorporates multisensory instructional strategies, including auditory input, visual scaffolding, and verbal or kinaesthetic responses, consistent with multimodal learning principles [4,5]. Instruction is delivered through a combination of whole-class and small-group activities to ensure consistent instructional exposure while maintaining manageable observation conditions.

The phase-based structure serves a dual purpose by providing pedagogical sequencing and establishing clearly defined observation contexts, thereby enabling systematic documentation of learner behaviours across instructional stages.

### Implementation protocol

The protocol was implemented over eight consecutive weeks (40 instructional hours) with a total sample of 93 Grade 1 learners across government, matriculation, and CBSE schools in Tamil Nadu. Class sizes ranged from approximately 25 to 35 learners, depending on the participating school. Instruction was delivered during routine classroom teaching using a structured multisensory programme organised into four sequential phases corresponding to the Listening, Speaking, Reading, and Writing (LSRW) domains. The method was designed to integrate classroom instruction with real-time behavioural observation without disrupting normal teaching activities.

Each instructional phase incorporated developmentally appropriate multisensory activities using auditory, visual, verbal, and kinaesthetic learning experiences. Activities included story listening, sound–word matching, picture description, guided verbal interaction, letter–sound association, word recognition, tracing, copying, and simple independent writing. These activities served both instructional and observational purposes, allowing learner behaviours to be documented within authentic classroom contexts rather than separate assessment sessions.

Instruction followed a standardised sequence during each activity:

Teacher introduction and task explanation (2–5 min)

Guided demonstration (approximately 5 min)

Learner practice (10–15 min)

Concurrent classroom observation during learner practice

Immediate rubric-based scoring (approximately 1 min per learner)

Transition to the subsequent activity

Whole-class instruction was used during demonstrations to ensure uniform exposure to learning activities. During learner practice, approximately 8–12 learners were observed consecutively during each activity while the remaining learners participated in the same instructional task. This number was selected to allow accurate real-time behavioural observation while ensuring that all learners were observed repeatedly across the programme. Observation order rotated systematically across activities and sessions so that every learner was observed repeatedly across all four LSRW domains during the eight-week programme. This rotation balanced observation workload while maintaining consistency and minimising disruption to classroom instruction.

Observed behaviours were documented using predefined behavioural indicators aligned with the instructional objectives of each language domain, including attention to input, response accuracy, participation, task completion, and independence. Immediately following each observation cycle, learner performance was classified using the three-level developmental rubric (Emerging, Developing, or Secure) based exclusively on directly observable classroom behaviours. Scores were recorded using a standardised spreadsheet template containing learner identifiers, instructional phase, activity context, observed behaviour, and developmental level.

Implementation fidelity was supported through a fixed instructional sequence, standardised observation boundaries, predefined behavioural descriptors, immediate post-activity scoring, and uniform data-recording procedures across all participating classrooms. These procedural controls were incorporated to promote consistency and facilitate replication of the method in comparable early primary ELT settings while preserving ecological validity.

### Classroom activities

Activities are embedded within routine classroom instruction and are developmentally appropriate for early primary learners. Listening tasks include story-based input, sound–word matching, and instruction-following activities. Speaking tasks involve guided responses, picture description, and short verbal interactions. Reading activities focus on letter–sound correspondence, word recognition, and visual tracking, while writing tasks include tracing, copying, and simple independent production.

Importantly, these activities are not designed as formal assessments but as instructional contexts within which observable behaviours can be systematically recorded. This ensures that data collection does not disrupt instructional flow and maintains ecological validity.

### Observation approach

Observation is conducted concurrently with instruction using predefined behavioural indicators, including attention to input, response accuracy, participation, task completion, and independence. These indicators are aligned with task requirements within each instructional phase.

Observation boundaries are operationalised by defining discrete instructional activities as observation units. Each observation cycle corresponds to a single activity block, during which behavioural indicators are documented. In larger classrooms, learners are observed in rotating subsets to maintain scoring accuracy and feasibility. The observer maintains a consistent physical position within the classroom to ensure visibility and procedural consistency.

This observation approach is informed by established classroom observation protocols, which demonstrate that real-time behavioural coding can be systematically implemented within naturalistic instructional settings [14,26].

### Rubric-based scoring

A three-level developmental rubric is used to categorise observed behaviours:

Emerging: Limited, inconsistent, or heavily supported performance

Developing: Partial task completion with intermittent support

Secure: Consistent and independent task performance

A three-level developmental rubric (Emerging, Developing, and Secure) was used to categorise observed behaviours across the four LSRW domains. While the developmental levels remained conceptually consistent, domain-specific behavioural descriptors were developed for listening, speaking, reading, and writing to improve scoring consistency and facilitate replication. Scoring was based exclusively on directly observable learner behaviours during instructional activities rather than curriculum mastery, academic achievement, or peer comparison. The domain-specific descriptors are presented in Table 3.

### Sample classroom observation and scoring example

Table 4 illustrates how the domain-specific scoring descriptors presented in Table 3 are applied during routine classroom observation. During each observation cycle, the observer records the instructional context, documents observable learner behaviours, and assigns the appropriate developmental level using the predefined rubric. The template serves as an illustrative example and may be adapted to different classroom activities while maintaining the same observation procedures and scoring criteria.

### Timing and scoring procedure

Scoring is conducted immediately following each activity block to minimise retrospective bias. Each observation and scoring cycle require approximately 1 min per learner, enabling consistent data collection during regular classroom time without disrupting teaching activities. This efficiency supports feasibility in low-resource classroom settings.

### Data recording and management

Each learner is assigned an anonymised identifier. Observations are recorded using standardised spreadsheet templates, with separate columns for each LSRW domain and observation point. The resulting dataset is ordinal and intended for descriptive documentation and methodological replication rather than psychometric analysis.

No composite or weighted scores are generated, as the method is designed to capture domain-specific behavioural patterns rather than produce aggregated performance indices.

### Progression across phases

Progression through instructional phases follows a predetermined sequence rather than individual mastery criteria. This ensures consistency of implementation across learners and classrooms, facilitating comparability and replication.

### Implementation requirements

The method requires minimal resources, including standard classroom materials such as flashcards, worksheets, and visual aids. No specialised software or assessment instruments are required. Classroom observations are recorded using the standardised spreadsheet template provided as supplementary Microsoft Excel material, which may also be used with Google Sheets or other compatible spreadsheet software. Implementation can be carried out by classroom teachers or researchers following brief familiarisation with the observation framework, domain-specific rubric descriptors, observation procedures, and spreadsheet-based recording system.

### Replication and adaptation

The method is designed for replication in early primary ELT contexts. While instructional materials may be adapted to local curricula, the core methodological components—phase-based instruction, real-time observation, rubric-based scoring, and structured data recording—should be retained to preserve methodological integrity.

## Method validation

Method validation focused on implementation feasibility, procedural consistency, and practical applicability, consistent with the methodological purpose of the study. The Multisensory LSRW Classroom Observation Method (M-LCOM) was implemented over eight weeks (40 instructional hours) during routine classroom sessions, ensuring ecological validity by embedding observation within authentic instructional settings rather than controlled experimental conditions.

Feasibility was demonstrated through the integration of observation and scoring into routine classroom teaching. Each observation cycle required approximately one minute per learner and was completed immediately following each instructional activity, enabling systematic data collection without interrupting teaching or requiring additional instructional time or specialised resources.

Procedural consistency was supported through predefined behavioural indicators, structured observation units, a standardised three-level developmental rubric, and a fixed phase-based instructional sequence applied uniformly across all instructional phases. These procedural controls ensured comparable observation conditions across learners and sessions while reducing variability associated with differences in task exposure.

**Observer preparation and calibration**. Before classroom implementation, the observer completed structured familiarisation with the observation protocol using representative classroom scenarios and pilot instructional activities. Behavioural indicators, observation boundaries, and rubric descriptors were reviewed repeatedly until scoring decisions could be applied consistently across all four LSRW domains. During implementation, identical observation procedures, scoring criteria, instructional sequences, and standardised recording formats were maintained across all sessions. Although formal inter-rater reliability was beyond the scope of this methodological study, these calibration procedures were incorporated to minimise observer variability and promote procedural consistency.

The validation approach draws upon established principles from structured classroom observation protocols, which demonstrate that real-time behavioural coding can produce consistent and replicable data when supported by clearly defined behavioural indicators and scoring criteria [14,15,26]. Although such protocols frequently employ multiple observers, the present method adapts these principles to a single-observer classroom context to prioritise feasibility while maintaining systematic observation procedures.

Data completeness was monitored throughout implementation. Missing observations occurred primarily because of learner absence or temporary inaccessibility during observation cycles and were treated as inherent features of classroom-based data collection rather than methodological errors. No imputation procedures were applied because the method is intended for descriptive documentation rather than inferential analysis.

Overall, the validation findings indicate that the method can be implemented consistently under routine classroom conditions, producing systematic documentation of learner behaviours during authentic instructional activities. The method is therefore suitable for replication in early primary ELT contexts where ecological validity and practical feasibility are essential.

The purpose of this validation was to demonstrate implementation feasibility, procedural consistency, and practical applicability rather than establish psychometric reliability or construct validity. Consequently, the findings should be interpreted as evidence that the method can be implemented systematically in authentic classroom settings, while formal evaluations of inter-rater reliability, construct validity, and other psychometric properties remain priorities for future research.

## Limitations

The present method adopts a single-observer design to prioritise feasibility and ecological validity within natural classroom environments. Although multi-observer designs are commonly employed in structured observation systems to establish inter-rater reliability, such approaches often require extensive training, time, and logistical resources that may not be feasible in low-resource educational contexts [14]. The current design, therefore, represents a pragmatic trade-off between methodological rigor and practical applicability.

The absence of an inter-rater reliability assessment is acknowledged as a limitation. Single-observer scoring may introduce bias in the interpretation of behavioural indicators. However, the use of predefined observation criteria, standardised rubric descriptors, and structured observation cycles was intended to minimise subjective variability. Future applications of the method may incorporate inter-rater agreement procedures, observer calibration protocols, and video-assisted validation to enhance reliability.

The observational rubric is non-standardised and context-specific, limiting its applicability for psychometric generalisation or comparison across studies. The method is not intended to replace formal assessment instruments but to complement them by providing ecologically valid documentation of observable learning behaviours during routine instruction.

Additionally, while the method is designed for adaptability across contexts, modifications to instructional materials and rubric descriptors may be necessary when applied to different age groups, curriculum frameworks, or classroom environments. Such adaptations should preserve the core methodological principles—phase-based instruction, real-time observation, and structured documentation—to maintain consistency.

Future studies should evaluate inter-rater agreement using multiple observers, video-assisted coding, or formal reliability statistics to further strengthen the methodological robustness of the observation framework Table 1, Table 2.

**Table 1:** Eight-week instructional implementation schedule.

Week	Phase	Domain	Hours
1–2	Phase I	Listening	10
3–4	Phase II	Speaking	10
5–6	Phase III	Reading	10
7–8	Phase IV	Writing	10

**Table 2:** Overview of instructional phases and observation focus.

Phase	Language skill	Example classroom activities	Primary observation focus
Phase I	Listening	Story listening, sound–word matching, instruction-following	Attention to input, response accuracy, task completion
Phase II	Speaking	Guided responses, picture descriptions, short verbal interactions	Participation, response clarity, independence
Phase III	Reading	Letter–sound association, word recognition, visual tracking	Accuracy, sustained engagement, task completion
Phase IV	Writing	Tracing, copying, simple independent writing	Motor control, task completion, independence

**Table 3:** Three-level developmental rubric structure.

Level	Descriptor	Observable indicators	Listening	Speaking	Reading	Writing
Emerging	Limited or inconsistent task performance requiring substantial support	Does not respond independently; requires repeated prompts; incomplete or inaccurate task execution	Requires repeated verbal prompts or visual cues to follow oral instructions.	Produces isolated words or no verbal response without continuous prompting.	Recognises few letters or words and requires substantial teacher support.	Requires repeated hand guidance or prompts and is unable to complete tracing or writing independently.
Developing	Partial task completion with intermittent support	Responds with prompts; demonstrates partial accuracy; completes task with assistance	Responds correctly after one or more prompts and follows simple instructions with occasional support.	Produces short words or phrases with occasional prompting.	Reads familiar letters or words with visual or verbal support.	Completes tracing or copying tasks with occasional verbal or visual assistance.
Secure	Consistent and independent task performance	Responds independently; demonstrates accurate and complete task execution; maintains engagement throughout activity	Follows oral instructions accurately and independently throughout the activity.	Produces clear and appropriate verbal responses independently using words or short sentences.	Reads target letters, words, or simple sentences accurately without teacher support.	Independently traces, copies, or writes the required letters, words, or simple sentences accurately.

**Table 4:** Sample classroom observation and scoring template.

Learner ID	LSRW domain	Activity context	Observed behaviour	Score level
S01	Listening	Sound–word matching	Identified the correct word after one verbal instruction and completed the task independently	Secure
S02	Listening	Sound–word matching	Responded correctly after repeated verbal prompts.	Developing
S03	Speaking	Picture description	Produced a clear verbal response using a short phrase.	Secure
S04	Reading	Word recognition	Recognised the target word with visual support from the teacher	Developing
S05	Writing	Letter tracing	Required repeated hand guidance and did not complete the task independently	Emerging

## Ethics statements

Human subjects:

The study was conducted in a school-based learning setting and focused on the routine teaching practices. Institutional permission was obtained from the participating school authorities before implementation. Written informed consent was obtained from the parents or legal guardians of all participating learners. Participation was voluntary, and no personally identifiable information was collected. All data were anonymised and used solely for academic research purposes. The study involved minimal risk and did not include any video or audio recording of learners.

## CRediT author statement

Srini Sandhya M: Conceptualization, Methodology, Data curation, Investigation, Writing – original draft.

Dr. Aravind B. R.: Supervision, Method validation, Writing – review & editing.

## Funding

This research did not receive any specific grant from funding agencies in the public, commercial, or not-for-profit sectors.

## Declaration of generative AI and AI-assisted technologies in the manuscript preparation process

During the preparation of this manuscript, the authors used Grammarly solely for language refinement. No AI tools were used for data analysis, interpretation, or generation of scientific content. All outputs were critically reviewed and edited by the authors. The authors take full responsibility for the integrity and accuracy of the work.

## Declaration of competing interest

The authors declare that they have no known competing financial interests or personal relationships that could have appeared to influence the work reported in this paper.

## Data Availability

Data will be available on request for academic purposes.
